# HDV+HBV hepatitis reactivation due to immunossuppressive therapy for hematological malignancies – an increasingly complex challenge

**DOI:** 10.1186/1471-2334-13-S1-O24

**Published:** 2013-12-16

**Authors:** Violeta Molagic, Raluca Mihăilescu, Cristina Popescu, Cătălin Tilişcan, Mihaela Rădulescu, Raluca Năstase, Ruxandra Moroti, Viorica Poghirc, Victoria Aramă

**Affiliations:** 1 National Institute for Infectious Diseases “Prof. Dr. Matei Balş”, Bucharest, Romania; 2 Carol Davila University of Medicine and Pharmacy, Bucharest, Romania

## Background

Hepatitis B virus reactivation (HBV-R) is becoming an increasingly concerning issue as monoclonal antibody therapy has become widely used for chronic lymphoproliferations. HBV-R can also occur in HBsAg negative patients who have only HBcAb positive (occult HBV infection).

We present a non-consecutive HBV-R case series report. Our series consisted of both HBsAg positive patients and patients with occult HBV infection, who received monoclonal antibody therapy. We present the most important demographic, clinical and immune-virological features of HBV-R in these patients.

## Case report

We also include the case of a 62 year old male patient with a previously HBs Ag negative, HBcAb positive status who was diagnosed with small B cell non Hodgkin lymphoma (NHL). The liver function tests were normal. The patient started cyclophosphamide-adriamycin-vincristine-prednisone+rituximab cycles and preemptive therapy with lamivudine 100 mg/day for HBV-R. After 5 cycles the patient developed asthenia and anorexia. He had increased serum aminotransferase (x10 ULN) levels and low platelet count. He tested negative for HAV, HCV, EBV, CMV markers and positive for HBV (positive HBsAg, HBcAb and HBeAb), with HBV-DNA of 79 UI/mL. The patient had HDV total antibodies and high viral load: HDV-RNA = 555,000,000 geq/mL. He was diagnosed with hepatitis HDV/HBV reactivation due to chemotherapy for B cell-NHL. Although NHL was in partial remission, the chemotherapy and lamivudine were stopped. He started pegIFN alpha-2a therapy when ALT values were almost within normal range.

## Conclusion

The problem of HBV/HDV hepatitis reactivation in patients receiving immunosuppressive treatment is insufficiently known. Preemptive antiviral therapy prior to monoclonal antibody administration may be crucial for preventing HBV-R even in the presence of occult HBV infection. It is important to draw attention to the possibility of HDV reactivation, as an occult delta virus infection may be present even in persons with occult HBV infection.

